# Supplementary material for: Government spending shocks and default risk in emerging markets
Source: PLoS One. 2023 Jul 20;18(7):e0288802. doi: 10.1371/journal.pone.0288802 (PMC10358995; doi:10.1371/journal.pone.0288802)
Supplement: S1 Appendix — (PDF) [file pone.0288802.s001.pdf]

Appendix A Computation

The model is solved using the discrete state-space method. The space for debt is discretized into 300 grid points with a lower bound of 0. The upper bound is set to be 0.1 and never binds along the equilibrium paths.

The solution algorithm consists of an inner loop and an outer looper:

- Inner loop: value function iteration. Given the price function q^0 in the outer loop, solve for the optimal policies in equations (2) an (3) using initial values V^0_{pay}, V^0_{def}, and $V^0 = \max\left\{V^0_{pay}, V^0_{def}\right\}$. Then obtain new values V^1_{pay}, V^1_{def}, and $V^1 = \max\left\{V^1_{pay}, V^1_{def}\right\}$. Update V^0_{pay}, V^0_{def}, and V^0 by V^1_{pay}, V^1_{def}, and V^1 until convergence.
- Outer loop: price function iteration. Using the default policy D^0 obtained in the inner loop, update the price q^0 by $q^1 = \frac{E\left[1-D^0\right]}{1+r^*}$ until convergence.

After the model has been solved, find the steady state in which the sovereign borrows the same amount of debt in each period. Then feed the path of government spending into the model and obtain the impulse responses.

Appendix B Data

Table 1: External Default/Restructuring: 1800-2010.

Country	Episodes	Frequency
Argentina	1827-1857; 1890-1893; 1951; 1956-1965;	7
	1982-1993; 1989; 2001-2005	
Brazil	1828-1834; 1898-1901; 1902-1910; 1914-1919;	9
	1931-1933; 1937-1943; 1961; 1964; 1983-1990	
Bulgaria	1932; 1990	2
Chile	1826-1842; 1880-1883; 1931-1947; 1961;	9
	1963; 1965; 1972; 1974-1975; 1983-1990	
Colombia	1826-1845; 1850-1861; 1873; 1880-1896;	7
	1900-1904; 1932-1934; 1935-1944	
Croatia	1993-1996	1
Ecuador	1826-1845; 1868-1890; 1894-1898; 1900-1904; 1906-1911;	10
	1914-1924; 1929-1954; 1982-1995; 1999-2000; 2008	
El Salvador	1828-1860; 1898; 1921-1922; 1932-1935; 1938-1946	5
Hungary	1932-1937; 1941-1967	2
Mexico	1828-1830; 1833-1841; 1844-1850; 1854-1864;	8
	1866-1885; 1914-1922; 1928-1942; 1982-1990	
Peru	1826-1848; 1876-1889; 1931-1951; 1969;	8
	1976; 1978; 1980; 1984-1997	
Poland	1932-1939; 1940-1952; 1981-1994	3
South Africa	1985-1987; 1989; 1993	3
Thailand	1997-1998 "near"	0
Turkey	1876-1881; 1915-1928; 1931-1932; 1940-1943;	8
	1959; 1965; 1978-1979; 1982; 2000-2001 "near"	
Uruguay	1876-1878; 1891; 1915-1921; 1932-1938; 1965;	9
	1983-1985; 1987; 1990-1991; 2003	

Data source: Reinhart, C. M. and Rogoff, K. S. (2011), "From financial crash to debt crisis," *American Economic Review*, 101(5), 1676-1706.

Table 2: Episodes of De-Facto Fixed and Flexible Exchange Rates: 1993Q1-2010Q4.

Country	Fixed	Flexible
Argentina	1993Q1-2001Q4; 2007Q2-2010Q4	2002Q1-2007Q1
Brazil	1994Q3-1999Q1	1993Q1-1994Q2; 1999Q2-2010Q4
Bulgaria	1997Q1-2010Q4	1993Q1-1996Q4
Chile	n.a.	1993Q1-2010Q4
Colombia	n.a.	1993Q1-2010Q4
Croatia	1994Q4-2010Q4	1993Q4-1994Q3
Ecuador	1997Q2-1997Q3; 2000Q2-2010Q4	1993Q1-1997Q1; 1997Q4-2000Q1
El Salvador	1993Q1-2010Q4	n.a.
Hungary	1994Q3-1999Q1	1993Q1-1994Q2; 1999Q2-2010Q4
Lithuania	1995Q2-2010Q4	1993Q1-1995Q1
Malaysia	1993Q1-1997Q3; 1998Q4-2010Q4	1997Q4-1998Q3
Mexico	1993Q1-1994Q4	1995Q1-2010Q4
Peru	1994Q1-2010Q4	1993Q1-1993Q4
Poland	n.a.	1993Q1-2010Q4
South Africa	n.a.	1993Q1-2010Q4
Thailand	1993Q1-1997Q2; 1999Q4-2010Q4	1997Q3-1999Q3
Turkey	n.a.	1993Q1-2010Q4
Uruguay	1995Q4-2001Q4; 2005Q3-2007Q4	1993Q1-1995Q3; 2002Q1-2005Q2;
		2008Q1-2010Q4

Data sources: Ilzetzki, E., Mendoza, E. G., and Végh, C. A. (2013), "How big (small?) are fiscal multipliers?" *Journal of Monetary Economics*, 60(2), 239-254.
Ilzetzki, E., Reinhart, C. M., and Rogoff, K. S. (2019)., "Exchange arrangements entering the twenty-first century: Which anchor will hold?" *Quarterly Journal of Economics*, 134(2), 599-646.
