# Supplementary material for: Government spending shocks and default risk in emerging markets
Source: PLoS One. 2023 Jul 20;18(7):e0288802. doi: 10.1371/journal.pone.0288802 (PMC10358995; doi:10.1371/journal.pone.0288802)
Supplement: Attachment — Submitted filename: Response_to_Reviewers.pdf [file pone.0288802.s004.pdf]

We would like to thank the referees for their very helpful comments and suggestions that can help us to improve the paper substantially. We have revised the paper according to these comments and suggestions.

The revisions made in response to each referee's comments are as follows.

Response to reviewer 1's comments

Comment 1: "*A gap has been underlined to justify the study but this contribution could have been written in a better way.*"

Thank you for the recommendation. We have added the following two paragraphs to the Introduction section so as to demonstrate this paper's contribution.

"This paper has two main contributions. First, models of sovereign default, based on the seminal framework in Eaton and Gersovitz (1981) and later extended by Aguiar and Gopinath (2006), Arellano (2008), and Arellano, Mateos-Planas, and Ríos-Rull (2023), typically abstract from fiscal policy. We contribute to this literature by adding government spending shocks to a sovereign default model. In this sense, our paper is most related to Espino et al. (2020), who investigate optimal fiscal and monetary policies for emerging markets in a sovereign default model. Our paper differs from Espino et al. (2020) notably by building an econometrics model and carrying out empirical analysis."

"Second, researchers have showed that government spending is usually less effective in stimulating output in developing countries than in advanced countries (Ilzetzki, Mendoza, and Végh, 2013; Sheremirov and Spirovska, 2022). Some obstacles preventing fiscal policy to be effective in developing countries include the procyclicality of fiscal policy (Talvi and Vegh, 2005), foreign aid financing of government spending (Kraay, 2012, 2014), and low marginal investment efficiency (Shen, Yang, and Zanna, 2018). We contribute to this literature by showing that increased default risk is also a factor that could weaken the stimulus effect of government spending in the long run."

Comment 2: "*Relevant literature has been incorporated but there is no recent article. The literature is not explained well so it is hard for a reader to understand the importance of the studied variables and relationships between them.*"

Thank you for this comment. We have rewritten the Introduction section with reference to recent articles. We have also written a separate section for literature review (Section 2 in the revised manuscript).

Comment 3: "*The results are provided but without explanation or discussion. No implications have been given to show how these findings can be utilised by economic and public policy makers, teachers and researchers.*"

Thank you for the suggestion. We have added discussions in the Introduction and Conclusion sections. In the Conclusion section, we have added the following paragraph about policy implications.

"Fiscal polices in emerging economies tend to be procyclical (Kaminsky, Reinhart, and Végh, 2004; Talvi and Vegh, 2005). Developing countries tend to spend more in good times and less in bad times. The findings of this paper have important implications for policymakers. A fiscal stimulus temporarily boosts GDP. However, as a nation's sovereign debt piles up, it has to pay a higher interest rate when borrowing from abroad, since the default risk is higher. This could exert a negative impact on the nation's economic activities in the long run. This external debt channel weakens the usefulness of government spending. If developing countries could instead save in good times, then it would attenuate the negative impact of the external debt channel. In this way, fiscal polices could be more effective."

Comment 4: "*The conclusion is weak.*"

Thank you for raising this point. In addition to adding policy implications, we have also added the following paragraph on limitations and future research directions.

"The current paper does not consider the financing method of government spending in developing countries. It is suggested for future researchers to distinguish among tax financed spending, debt financed spending, and money financed spending. Moreover, since government spending multiplier depends on the degree of economic development (Sheremirov and Spirovska, 2022), it will be useful for future researchers to distinguish among low-income economies, lower-middle-income economies, and upper-middle-income economies, as data become available. Finally, it would be important to explore the interplay between monetary and fiscal policies in a sovereign default model. This direction is also left for future research."

Response to reviewer 2's comments

Comment 1 Abstract:

"*a) Abstract should be more concrete and therefore needs realignment.*"

Thank you for the comment. We have rewritten the abstract.

"*b) The keywords are more or less repetition of the research title. It is suggested to revisit the keywords.*"

Thank you for the suggestion. We have revised the keywords.

Comment 2 Introduction:
"*a) The paragraph 1 lacks references. All the statements, facts and rationale put forth should be supported with proper in text citations.*"

Thank you for the reminder. We have rewritten paragraph 1 as follows.

"In order to combat the coronavirus pandemic, governments all over the world have implemented fiscal actions, including additional spending, tax cuts, and loans and capital injections by the public sector (IMF, 2021). On the one hand, fiscal support in response to the pandemic has prevented more severe economic recessions. On the other hand, such support has led to larger government deficits and debt. In 2020, average overall deficit as a share of GDP is 11.7 percent for advanced economies, 9.8 percent for emerging market economies, and 5.5 percent for low-income developing countries (IMF, 2021). Moreover, average public debt worldwide has reached an unprecedented 97 percent of GDP (IMF, 2021). Countries such as Argentina, Ecuador, Ethiopia, and Lebanon defaulted on their debts. This paper studies the effects of government spending on default risk in emerging economies. "

"*b) The contribution of the research is missing. It is recommended to add the contributions and justify your contribution. For example, review the following papers and gain insight:...*"

Thank you very much for providing references. We have added the following two paragraphs to the Introduction section so as to demonstrate this paper's contribution.

"This paper has two main contributions. First, models of sovereign default, based on the seminal framework in Eaton and Gersovitz (1981) and later extended by Aguiar and Gopinath (2006), Arellano (2008), and Arellano, Mateos-Planas, and Ríos-Rull (2023), typically abstract from fiscal policy. We contribute to this literature by adding government spending shocks to a sovereign default model. In this sense, our paper is most related to Espino et al. (2020), who investigate optimal fiscal and monetary policies for emerging markets in a sovereign default model. Our paper differs from Espino et al. (2020) notably by building an econometrics model and carrying out empirical analysis."

"Second, researchers have showed that government spending is usually less effective in stimulating output in developing countries than in advanced countries (Ilzetzki, Mendoza, and Végh, 2013; Sheremirov and Spirovska, 2022). Some obstacles preventing fiscal policy to be effective in developing countries include the procyclicality of fiscal policy (Talvi and Vegh, 2005), foreign aid financing of government spending (Kraay, 2012, 2014), and low marginal investment efficiency (Shen, Yang, and Zanna, 2018). We contribute to this literature by showing that increased default risk is also a factor that could weaken the stimulus effect of

government spending in the long run."

Comment 3 Theoretical background and hypothesis development: "*The introduction and literature review section are merged as one. It is fine although recommend to divide it under two parts. Moreover, there is a need for more recent studies ranging from 2018-2023 to support the study hypothesis properly. The entire study is too scanty and the related literature is not exhausted.*"
"*a) The first part of introduction is supported with many facts and figures but yet again there is quite old reference to support the statements.*"

Thank you for raising this point. We have added recent data and facts. The Introduction section has been revised substantially.
"*b) The literature review is also weak. The hypothesis statements should be added and explained well. The theoretical connections should be established to justify the research variables and proposed hypothesis. Moreover, update references are required.*"

We have added a separate section for literature review (Section 2 in the revised paper). Recent papers have been included.

Following the practice in macroeconomics, we first build a theoretical DSGE model and obtain predictions from model simulation (Section 3). We then develop an econometrics model and obtain empirical results (Section 4). The empirical results confirm the theoretical predictions. To emphasize this point, we have also revised the abstract and the Conclusion section. In the abstract, we have added this sentence: "The empirical results confirm the theoretical predictions from the general equilibrium model." In the Conclusion section, we have the following statement.

"In this paper, we have built a general equilibrium small open economy model that features a higher sovereign default risk in response to positive government spending shocks. VAR evidence from a panel of 18 emerging countries shows that the sovereign spread increases following a positive government spending shock, confirming the theoretical predictions from the general equilibrium model."

Comment 4 Data and methodology: "*The econometric modelling presented is good. However, it is suggested proper reasoning for opting VAR should be provided with references.*"

Thank you for the recommendation. The reasoning for opting VAR is provided in the following paragraphs.

"We then carry out empirical analysis and identify government spending shocks using structural vector autoregression (SVAR). There are two main strategies of identifying gov-

ernment spending shocks with aggregate data: SVAR analysis as in Blanchard and Perotti (2002) and the narrative approach developed by Ramey and Shapiro (1998). Blanchard and Perotti (2002) identify fiscal shocks by assuming that government spending is predetermined within one quarter so that it does not respond to other structural shocks contemporaneously. Ramey and Shapiro (1998) add a military build-up dummy variable in the autoregressive model to isolate an exogenous component of government spending. The two methods usually produce different responses to government spending shocks. Specifically, the first approach finds that consumption and real wages rise after a positive government spending shock, consistent with the predictions of New Keynesian models. In contrast, the second approach generates a fall in the two variables which is often consistent with neoclassical models. In this paper, we use a panel VAR to identify government spending shocks. The reason for using a panel VAR is twofold: first, the narrative approach cannot be applied to the panel of developing countries studied in this paper, since it requires that wars be extraterritorial, which is not the case for most of the developing countries; second, the inception of the EMBI Global spread data varies from 1993Q4 to 2009Q4 in different countries, making individual VAR analysis impractical in many countries."

"The VAR approach has been criticized for ignoring the "anticipation effect": the VAR-identified fiscal shocks may have been expected by the private sector, who will take action even before the spending is realized. Ilzetzki, Mendoza, and Végh (2013) show in their online appendix that this is not the case in developing countries. They argue that agents in developing countries could not have a good estimate of fiscal shocks because fiscal shocks are sufficiently volatile in developing countries. The authors compare the central bank's estimation errors of government consumption with the VAR residuals in four developing countries. Central bank errors are computed as the difference between final statistics on government consumption and the central bank's estimate in the quarter following the time of the government expenditure. The authors find a strong correlation between central bank errors and VAR residuals."

Comment 5 Results and Discussion:"*There is hardly any referencing to support the results. The results are although quite significant and interesting. However, there needs to be proper discussion to support/reject the hypothesis made earlier.*"

Thank you for the recommendation. Please see our response to Comment 3.

Comment 6 Conclusion and Implications:

"*a) I would highly recommend inclusion of this section in the revised manuscript. You can*

review the following paper for this section..."

Thank you for the recommendation. In the Conclusion section, we have added the following paragraph about policy implications.

"Fiscal polices in emerging economies tend to be procyclical (Kaminsky, Reinhart, and Végh, 2004; Talvi and Vegh, 2005). Developing countries tend to spend more in good times and less in bad times. The findings of this paper have important implications for policy-makers. A fiscal stimulus temporarily boosts GDP. However, as a nation's sovereign debt piles up, it has to pay a higher interest rate when borrowing from abroad, since the default risk is higher. This could exert a negative impact on the nation's economic activities in the long run. This external debt channel weakens the usefulness of government spending. If developing countries could instead save in good times, then it would attenuate the negative impact of the external debt channel. In this way, fiscal polices could be more effective."

"*b) It is suggested to add one paragraph on study limitations and future research direction.*"

Thank you for the suggestion. We have added the following paragraph on limitations and future research directions.

"The current paper does not consider the financing method of government spending in developing countries. It is suggested for future researchers to distinguish among tax financed spending, debt financed spending, and money financed spending. Moreover, since government spending multiplier depends on the degree of economic development (Sheremirov and Spirovska, 2022), it will be useful for future researchers to distinguish among low-income economies, lower-middle-income economies, and upper-middle-income economies, as data become available. Finally, it would be important to explore the interplay between monetary and fiscal policies in a sovereign default model. This direction is also left for future research."

Comment 7: "*Last but not the least, there are many grammatical errors in the entire manuscript. It is highly recommended to review the document in detail. It is recommended to use professional reference and citation software so that paper seems well presented.*"

Thank you for the comment. We have made revisions. APA in-text citation has been utilized.

References

Aguiar, M. and Gopinath, G. (2006), "Defaultable debt, interest rates and the current account," *Journal of International Economics*, 69(1), 64-83.

Arellano, C. (2008), “Default risk and income fluctuations in emerging economies,” *American Economic Review*, 98(3), 690-712.

Arellano, C., Mateos-Planas, X., and Ríos-Rull, J. V. (2023), “Partial default,” *Journal of Political Economy*, forthcoming.

Blanchard, O. and Perotti, R. (2002), “An empirical characterization of the dynamic effects of changes in government spending and taxes on output,” *Quarterly Journal of Economics*, 117(4), 1329-1368.

Eaton, J. and Gersovitz, M. (1981), “Debt with potential repudiation: Theoretical and empirical analysis,” *Review of Economic Studies*, 48(2), 289-309.

Espino, E., Kozlowski, J., Martin, F. M., and Sanchez, J. M. (2020), “Seigniorage and sovereign default: The response of emerging markets to COVID-19,” *Federal Reserve Bank of St. Louis working paper.*

Ilzetzki, E., Mendoza, E. G., and Végh, C. A. (2013), “How big (small?) are fiscal multipliers?” *Journal of Monetary Economics*, 60(2), 239-254.

IMF (2021), “Fiscal monitor: A fair shot,” *International Monetary Fund*, April 2021.

Kaminsky, G. L., Reinhart, C. M., and Végh, C. A. (2004), “When it rains, it pours: Procyclical capital flows and macroeconomic policies,” *NBER Macroeconomics Annual*, 19, 11-53.

Kraay, A. (2012), “How large is the government spending multiplier? Evidence from World Bank lending,” *Quarterly Journal of Economics*, 127(2), 829-887.

Kraay, A. (2014), “Government spending multipliers in developing countries: evidence from lending by official creditors,” *American Economic Journal: Macroeconomics*, 6(4), 170-208.

Ramey, V. A. and Shapiro, M. D. (1998), “Costly capital reallocation and the effects of government spending,” *Carnegie-Rochester Conference Series on Public Policy*, 48, 145-194.

Shen, W., Yang, S. C. S., and Zanna, L. F. (2018)., “Government spending effects in low-income countries,” *Journal of Development Economics*, 133, 201-219.

Sheremirov, V. and Spirovska, S. (2022), “Fiscal multipliers in advanced and developing countries: Evidence from military spending,” *Journal of Public Economics*, 208, 104631.

Talvi, E. and Vegh, C. A. (2005), “Tax base variability and procyclical fiscal policy in developing countries,” *Journal of Development economics*, 78(1), 156-190.
