# Supplementary material for: Government spending shocks and default risk in emerging markets
Source: PLoS One. 2023 Jul 20;18(7):e0288802. doi: 10.1371/journal.pone.0288802 (PMC10358995; doi:10.1371/journal.pone.0288802)
Supplement: Attachment — Submitted filename: Plos One Review.docx [file pone.0288802.s003.docx]

**1) Abstract**

a) Abstract should be more concrete and therefore needs realignment.

a) The keywords are more or less repetition of the research title. It is suggested to revisit the keywords.

**2) Introduction:**

a) The paragraph 1 lacks references. All the statements, facts and rationale put forth should be supported with proper in text citations.

b) The contribution of the research is missing. It is recommended to add the contributions and justify your contribution. For example, review the following papers and gain insight:

i) Cai, L., Firdousi, S. F., Li, C., & Luo, Y. (2021). Inward foreign direct investment, outward foreign direct investment, and carbon dioxide emission intensity-threshold regression analysis based on interprovincial panel data. *Environmental Science and Pollution Research*, 1-14.

ii) Li, C., Firdousi, S. F., & Afzal, A. (2022). China’s Jinshan Yinshan sustainability evolutionary game equilibrium research under government and enterprises resource constraint dilemma. *Environmental Science and Pollution Research*, *29*(27), 41012-41036.

iii) Mirza, N., Umar, M., Afzal, A., & Firdousi, S. F. (2023). The role of fintech in promoting green finance, and profitability: Evidence from the banking sector in the euro zone. *Economic Analysis and Policy*, *78*, 33-40.

iv) Awais, M., Afzal, A., Firdousi, S., & Hasnaoui, A. (2023). Is fintech the new path to sustainable resource utilisation and economic development?. *Resources Policy*, *81*, 103309.

**3) Theoretical background and hypothesis development**

The introduction and literature review section are merged as one. It is fine although recommend to divide it under two parts. Moreover, there is a need for more recent studies ranging from 2018-2023 to support the study hypothesis properly. The entire study is too scanty and the related literature is not exhausted.

a) The first part of introduction is supported with many facts and figures but yet again there is quite old reference to support the statements.

b) The literature review is also weak. The hypothesis statements should be added and explained well. The theoretical connections should be established to justify the research variables and proposed hypothesis. Moreover, update references are required.

**4) Data and methodology**

The econometric modelling presented is good.  However, it is suggested proper reasoning for opting VAR should be provided with references.

**5) Results and Discussion**

a) There is hardly any referencing to support the results. The results are although quite significant and interesting. However, there needs to be proper discussion to support/reject the hypothesis made earlier.

**6) Conclusion and Implications**

a) I would highly recommend inclusion of this section in the revised manuscript. You can review the following paper for this section:

i) Li, C., Firdousi, S. F., & Afzal, A. (2022). China’s Jinshan Yinshan sustainability evolutionary game equilibrium research under government and enterprises resource constraint dilemma. *Environmental Science and Pollution Research*, *29*(27), 41012-41036.

ii) Afzal, A., Firdousi, S. F., Waqar, A., & Awais, M. (2022). The Influence of Internet Penetration on Poverty and Income Inequality. *Sage Open*, *12*(3), 21582440221116104.

b) It is suggested to add one paragraph on study limitations and future research direction.

Last but not the least, there are many grammatical errors in the entire manuscript. It is highly recommended to review the document in detail. It is recommended to use professional reference and citation software so that paper seems well presented.
